# Characterization of Pseudorange Errors in Hybrid LEO/MEO PNT System

**DOI:** 10.3390/s26144434

**Published:** 2026-07-13

**Authors:** Andrea Bianconi, Omar Morandi, Simone Morosi, Marco Dolfi

**Affiliations:** 1 Department of Information Engineering, University of Florence, 50139 Florence, Italy; andrea.bianconi@unifi.it (A.B.); simone.morosi@unifi.it (S.M.); 2 Department of Mathematics and Computer Science, University of Florence, 50139 Florence, Italy; omar.morandi@unifi.it

**Keywords:** GNSS, LEO constellations, hybrid positioning, atmosphere error, TLE error, clock bias, multipath errorr, PNT resilience

## Abstract

Integrating low Earth orbit (LEO) satellites into GNSS (Global Navigation Satellite System) MEO (Medium Earth Orbit) constellations enhances global positioning but faces technical bottlenecks. Through a 1D vehicular scenario simulation using MATLAB-based stochastic modeling of error sources and Least Squares estimation, MEO, LEO and hybrid configurations are evaluated, accounting for ionospheric, atmospheric and multipath errors. Results show that a single LEO satellite fails to improve accuracy due to the absence of onboard atomic clocks and the limitations of using Two-Line Elements (TLEs). However, a threshold of three LEO satellites significantly reduces positioning errors by approximately 35% through effective error averaging. The findings demonstrate that transitioning from TLEs to Precise Orbit Determination (POD) and clock stability are essential for LEO augmentation. These results provide an analytical framework for resilient PNT (Positioning, Navigation and Timing) applications, including autonomous driving and smart cities.

## 1. Introduction

Over the past few decades, satellite positioning has evolved into a fundamental pillar for a vast number of applications, ranging from land and maritime navigation to geodesy, environmental monitoring, autonomous vehicles and location-based services for the Internet of Things (IoT) [1]. Within modern society, the ability to obtain precise positioning and timing synchronization data is now indispensable, providing critical support to sectors such as maritime and air navigation, national power grid synchronization and the rapidly growing autonomous transport industry. Furthermore, the massive expansion of the IoT has created a pressing demand for high-precision and low-power localization services that existing infrastructures often struggle to meet consistently. To date, Global Navigation Satellite Systems (GNSSs), relying on Medium Earth Orbit (MEO) constellations, remain the most widely adopted solution for global positioning. This dominance is due to their planetary coverage, the relative ease of signal reception, the continuous evolution of existing constellations and the proliferation of low-cost systems capable of processing satellite signals. Despite their success, MEO systems have significant intrinsic limitations, particularly when operating in challenging environments [2,3]. Since MEO satellites are positioned at altitudes of approximately 20,000 km [4], the signal power reaching the Earth’s surface is extremely weak, making it highly vulnerable to severe attenuation and blockage by physical obstacles. Moreover, traditional GNSS remains susceptible to atmospheric delays (ionospheric and tropospheric), multipath effects and interferences—such as jamming and spoofing—which can compromise the overall integrity of the positioning solution [5,6,7]. In response to these growing challenges, both the scientific community and the industrial sector are shifting toward a new approach: low Earth orbit (LEO) constellations [4,8]. Originally designed for broadband communications, high-resolution Earth observation, and global internet services, LEO satellites offer unique structural advantages for positioning. Their proximity to Earth (typically between 350 and 2000 km [4,9]) ensures a significantly higher signal-to-noise ratio (SNR) compared to MEO systems, facilitating better signal penetration in indoor environments or in contexts where GNSS signals are heavily attenuated. Additionally, the high orbital velocity characteristic of LEO satellites results in a rapidly changing geometry; this reduces the convergence time for high-precision algorithms, such as Precise Point Positioning (PPP), and minimizes the duration of periods where the user lacks a direct line-of-sight (LOS) with the satellite [10,11,12].

In the field of positioning, LEO satellites can be used as an augmentation system for currently operational GNSS systems. There are two different possible approaches: Signal of Opportunity (SoO) and dedicated LEO PNT (Positioning, Navigation and Timing) systems [1]. In the SoO approach, the signals transmitted by the thousands of LEO satellites currently orbiting around the Earth are used opportunistically; these satellites involved in the process were not specifically designed for positioning, they do not transmit specific navigation signals, and, therefore, various measurements must be used to determine all the information essential for positioning algorithms to calculate the satellite’s and user’s position. This approach exploits existing broadband or communication mega-constellations such as SpaceX’s Starlink or Iridium [13], which do not inherently broadcast navigation data, because positioning is achieved opportunistically by tracking Doppler shifts or carrier phase variations. Conversely, in dedicated LEO-PNT systems, a mega-constellation of satellites, whose primary application area is the provision of positioning services, is designed and built. The signals that allow PNT measurements to be performed are managed by a payload designed for this specific purpose; for this reason, the signals, frequency band and antenna coverage are optimized with positioning as the primary objective. The built-in LEO-PNT approach would be advantageous in situations where GNSS signals are not available or blocked. Globally, this sector is making rapid strides at both national and international levels. Within Europe, key initiatives include the ESA LEO-PNT mission, which aims to deploy a low Earth orbit satellite system that operates in synergy with Galileo [14], as well as cutting-edge programs like European IRIS^2^ (Infrastructure for Resilience, Interconnectivity and Security by Satellite) and the SESAR frameworks (Single European Sky ATM Research) for air traffic management modernization; these require highly robust satellite communication channels (SATCOM) that integrate PNT functions to ensure ultra-secure 4D flight trajectory determination [15]. Looking further afield, highly active areas of research include US studies on the feasibility of an opportunistic system utilizing Starlink LEO satellites [16,17] and initiatives such as Pulsar, aimed at creating an autonomous LEO PNT navigation system, along with BeiDou LEO expansion in Asia [8]. This paper focuses specifically on the deployment of LEO satellites as an augmentation layer for traditional GNSS to realize a hybrid system. The practical value of integrating LEO satellites into traditional medium Earth orbit GNSS architectures lies in their ability to mitigate and overcome some of the structural vulnerabilities that current navigation systems are subject to in harsh environments. GNSS signals can become unusable in certain contexts, such as in urban environments with poor visibility or in the presence of intentional or unintentional interference, or indoors. LEO satellites benefit from significantly lower path loss due to their proximity to the Earth, typically operating between 350 and 2000 km from the Earth’s surface compared to over 20,000 km for MEO satellites. Furthermore, their proximity to the end user results in higher received signal strengths of up to 20–30 dB higher than standard GNSS [9], ensuring better performance in forests, urban canyons, and, in general, in those harsh situations where traditional GNSS signals would be too attenuated to be effectively used by the receiver. LEO satellites move at a speed approximately twice that of MEOs, resulting in rapid changes in the geometry of the mega-constellation [10]; this change is particularly useful in cases where the user has no satellites in sight or fewer than four. Since LEOs are faster than MEO satellites, the receiver experiences less line-of-sight loss and can return more quickly to an optimal condition to calculate its position. This aspect is particularly useful in urban environments where, due to the presence of tall buildings or skyscrapers, the GNSS signal can be blocked for a very long time. Consequently, the rapid change in geometry reduces the time during which an obstacle obstructs the line of sight and at the same time accelerates the decorrelation of atmospheric errors and multipath [10,18], allowing the receiver estimation filter to reach centimeter-level convergence within minutes or even seconds, successfully resolving the issue of slow filter convergence, which represents a serious problem in applications such as PPP [11,12]. Finally, thanks to the physical and geometric advantages of a hybrid LEO-MEO architecture, it is possible to solve the problems related to spoofing and jamming, while ensuring the continuity, precision and availability of the positioning service as required by safety-critical applications, such as autonomous driving and smart cities infrastructure.

This article provides a comprehensive exploration of the potential offered by LEO-based positioning, specifically quantifying the pseudorange errors across MEO, LEO and hybrid LEO-MEO constellations. The goal is to see whether LEO satellites can improve the performance of traditional MEO systems. The analysis systematically characterizes individual error sources, including ionospheric and tropospheric delays, as well as multipath effects, while addressing critical constraints that affect specifically LEO platforms, such as the reliance on Two-Line Elements (TLEs) for orbital determination and the inherent instability resulting from the absence of on-board atomic clocks. Furthermore, the research evaluates both the isolated impact of each disturbance and the total error, providing a comparative performance benchmark (Table 1). To evaluate the robustness of these architectures in non-optimal conditions, the analysis is extended to scenarios involving reduced constellations. Through rigorous error modeling—considering clock biases, orbital uncertainties (based on Two-Line Elements), atmospheric delays and multipath effects—and comparative simulations conducted in the MATLAB environment (Version 25.1, R2025a), this study demonstrates that the structural weaknesses of traditional MEO systems can be effectively overcome. To ensure a high degree of precision in isolating the impact of stochastic noise, the evaluation of positioning error is conducted within a vehicular operational scenario. By focusing on navigation along a predefined path, the analysis adopts a one-dimensional (1D) model, which reduces the dimensionality of the problem. This approach allows for a more agile investigation into how LEO signal characteristics influence error estimation along the longitudinal direction of travel, preventing the dilution of error across multiple coordinate axes and finding clearer performance benchmarks. However, it is important to emphasize that this simplification does not compromise the generality of the study; the implemented simulator remains inherently modular, ensuring that the results are scalable to 2D and 3D coordinate systems. Although the analysis focuses on a one-dimensional scenario constrained to a specific trajectory, the underlying simulation architecture developed in MATLAB is natively extensible to two- and three-dimensional systems; this is achieved by extending the spatial degrees of freedom within the least-squares estimation algorithm, as the simulation framework was specifically designed to accommodate such expansion. However, extending results obtained in one dimension to two- and three-dimensional positioning requires accounting for spatial anisotropy; indeed, in positioning systems, the vertical error component is inherently dominant over the horizontal planar components, as satellites can only be tracked by the receiver when they are above the horizon. Consequently, shifting from a 1D to a 3D configuration causes positioning uncertainty to evolve from a linear variance into a time-varying error ellipsoid, the parameters of which fluctuate due to multipath reflections and atmospheric dynamics. By adjusting the degrees of freedom within the simulation framework, the observation matrix projects pseudorange determination errors onto a two-dimensional plane or a three-dimensional volume, ensuring that the complex spatial correlations and anisotropic physical characteristics of multidimensional positioning systems are appropriately taken into account.

The ultimate objective is to pave the way for a new generation of localization services capable of maintaining high-integrity positioning in scenarios where signal degradation was previously considered inevitable.

The sections of this article are organized as follows: Section 2 describes the error sources affecting traditional GNSS and those specific to LEO satellites. Section 3 defines the operational scenario and the analysis methodology adopted. Section 4 details the mathematical modeling of atmospheric, multipath, TLE and clock error sources and includes simulation results for each error type. Section 5 presents the simulation results considering the overall error obtained by combining all sources of disturbances. Finally, Section 6 concludes the article and outlines future research directions.

## 2. Error Sources

In MEO systems, accurate positioning is constrained by three primary physical factors that degrade pseudorange measurements.

- Ionospheric delay: The ionosphere is the most significant source of error for GNSS signals. As a dispersive medium characterized by a high density of free electrons, it alters the signal propagation velocity. The delay is inversely proportional to the square of the signal frequency ($1/f^{2}$), affecting primarily single-frequency receivers [19].
- Tropospheric delay: Unlike the ionosphere, the troposphere is a non-dispersive medium influenced by local temperature, atmospheric pressure and water vapor content. The delay is highly dependent on local meteorological conditions and the air mass encountered along the signal path [3].
- Multipath effect: Multipath error occurs when the signal reaches the receiver via reflections from surrounding surfaces (buildings, terrain or obstacles) rather than through a direct LOS path. Due to the fixed position of MEO satellites relative to the Earth’s surface, the reflection geometry remains stationary over time. In urban canyon environments, this results in a static and persistent error, preventing the use of temporal statistical filters to remove it [6,9,20].

Unlike MEO satellites, LEO constellations operate in a highly dynamic environment with significant hardware and orbital constraints. The following sections analyze the primary error sources specific to LEO-PNT systems:

- Oscillator instability: Due to strict size, weight and power constraints, LEO satellites typically cannot carry traditional atomic clocks. LEOs often employ high-performance MEMSs (Micro-Electro-Mechanical Systems) or Chip-Scale Atomic Clocks (CSACs). While efficient, these oscillators lack the long-term stability of MEO atomic clocks, leading to unpredictable frequency drifts and pseudorange biases [21,22].
- Precise Orbit Determination (POD) and prediction: Unlike MEO satellites, LEO platforms typically do not broadcast native ephemerides or the full suite of orbital parameters required for direct pseudorange computation. Instead, orbital information is often retrieved through Two-Line Elements (TLEs) provided by organizations such as NORAD (North American Aerospace Defense Command) [16]. TLEs represent a standardized format for satellite orbital state vectors. However, their periodic update cycle, typically occurring only once or twice per day, is insufficient to track the highly dynamic trajectory of LEO satellites with high precision [23,24].
- Atmospheric and channel effects: LEO signal path presents unique advantages compared to GNSS:
  – Ionospheric mitigation: LEO satellites orbit within or just above the ionosphere (350–2000 km). Consequently, their signals traverse only a fraction of the Total Electron Content (TEC), resulting in a lower ionospheric delay compared to MEO signals [5,25].
  – Tropospheric decorrelation: The high angular velocity of LEOs allows for a faster decorrelation of the tropospheric wet delay, significantly reducing the convergence time for precise positioning.
  – Multipath and indoor penetration: LEO constellations provide a significantly higher signal-to-noise ratio (SNR) on the ground (up to 30 dB higher than MEO). This increased power enhances indoor penetration and signal robustness. Furthermore, the rapid motion of LEO satellites transforms static multipath into a wideband noise-like signal, which is more easily mitigated by receiver algorithms [9,18].

## 3. Materials and Methods

This section focuses on the integration of low Earth orbit satellites within a traditional 8-satellite Medium Earth Orbit constellation. The primary objective is to develop a multilayer architecture capable of compensating for inherent limitations and positioning errors of standard GNSS systems. The analysis is conducted through numerical simulations in the MATLAB environment using a synthetic data generation approach. The simulation framework evaluates positioning performance by modeling the uncertainty of pseudorange measurements.

The effectiveness of the localization system is evaluated by comparing three distinct orbital configurations:

- MEO scenario: the baseline reference representing conventional GNSS systems.
- LEO scenario: used to assess the specific potential of low-orbit constellations.
- Hybrid LEO+MEO scenario: aimed at quantifying the actual performance gain and benefits derived from integrating low-orbit satellites into the MEO segment.

**Figure 1:**
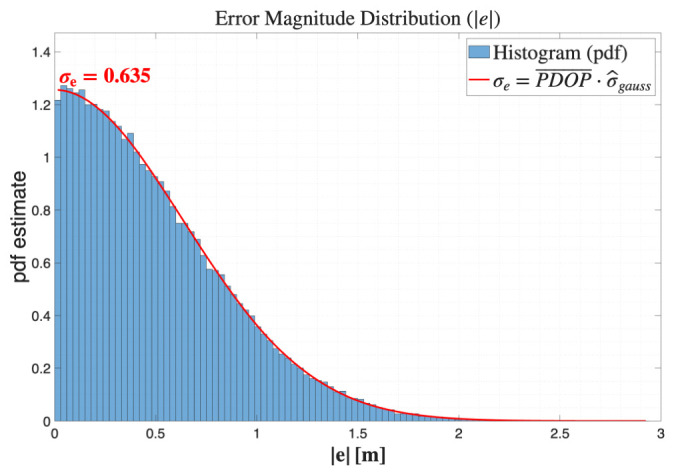
Positioning error Probability Density Function (PDF) and standard deviation ($\sigma_{e}=0.635$) for a 8-MEO constellation under a zero-mean Gaussian error scenario.

The Least Squares (LS) algorithm is used to estimate the receiver’s position [26]. Initially, a constellation composed of eight satellites in medium orbit is considered. For a receiver in a known location, the positioning error is evaluated in terms of standard deviation $\sigma_{e}$ resulting from the application of the least squares algorithm. To model the error committed in a GNSS measurement, a zero-mean Gaussian random error is superimposed on the pseudorange measurements (Figure 1). Although the purpose of this choice is to define an initial baseline for positioning error, specific models are employed in subsequent sections to realistically evaluate system performance by adequately characterizing the effects of various error sources. One of the objectives is to determine how $\sigma_{e}$ changes by evaluating how possible error sources can influence position determination and how the presence of any LEO satellite in the constellation succeeds or fails to mitigate the overall error in the final calculated position. For vehicular applications, navigation typically occurs along a known path, and the navigation problem can be effectively formulated as a one-dimensional constrained estimation along the predefined direction of travel of the receiver. Furthermore, to account for the simulation’s kinematic assumptions, the receiver is modeled as strictly stationary (velocity = 0 m/s) at a known location. This zero-velocity assumption is intentionally adopted to isolate the instantaneous positioning error, calculated as the difference between the receiver’s true known position and its estimated position, with the aim of evaluating how this error varies based on the integration of LEO satellite constellations. This assumption simplifies the scenario by neglecting dynamic error sources (such as receiver tracking loop jitter under acceleration and time-varying Doppler shifts), thereby ensuring that any observed improvements in the Probability Density Function (PDF) and the error standard deviation can be attributed solely to the characteristics of the constellations under examination. In this context, although the receiver’s position is determined for a single spatial degree of freedom, the geometry of the satellite constellation remains fully three-dimensional. Line-of-sight vectors are derived from the constellation’s observation matrix and mathematically projected onto the axis corresponding to the receiver’s trajectory; consequently, the standard deviation of the positioning error is evaluated along the axis of motion; however, this standard deviation is influenced by the 3D distribution of satellites within the considered constellation. Evaluating the standard deviation along this specific direction of motion—rather than using an overall 3D positioning error metric—is advantageous. As noted previously, a global 3D error metric is dominated by the vertical component, which is inherently larger than the horizontal components, making it impossible to effectively detect errors occurring in the horizontal plane. Therefore, simplifying the problem into a one-dimensional scenario (default horizontal and vertical reference angles set to 0°) allows an adequate analysis of the error component of interest. Finally, the necessary corrections for the errors considered are introduced in order to evaluate if it is possible to improve the accuracy of the determined position.

## 4. Results

**Figure 2:**
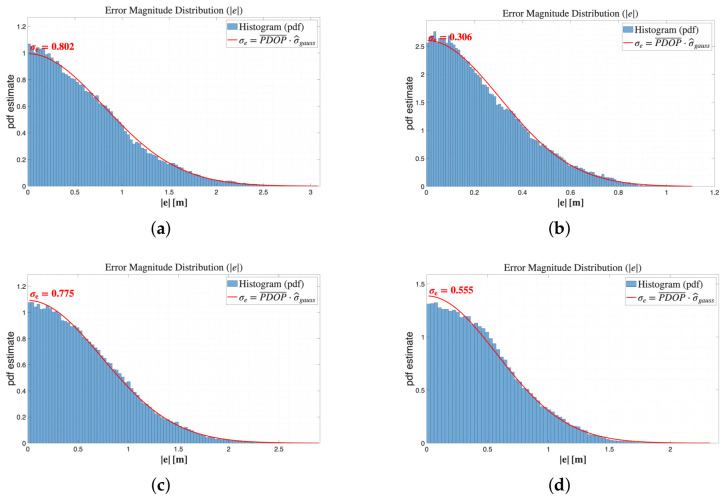
Positioning error PDFs under ionospheric delay: (**a**) 8-MEO configuration ($\sigma_{e}=0.802$), (**b**) 8-LEO configuration ($\sigma_{e}=0.306$), (**c**) hybrid 7-MEO and 1-LEO configuration ($\sigma_{e}=0.775$) and (**d**) hybrid 5-MEO and 3-LEO configuration ($\sigma_{e}=0.555$).

**Figure 3:**
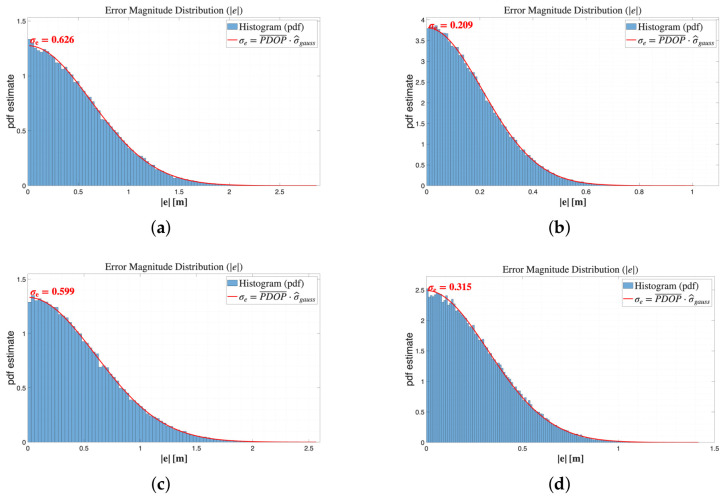
Positioning error PDFs under tropospheric delay: (**a**) 8-MEO configuration ($\sigma_{e}=0.626$), (**b**) 8-LEO configuration ($\sigma_{e}=0.209$), (**c**) hybrid 7-MEO and 1-LEO configuration ($\sigma_{e}=0.599$) and (**d**) hybrid 6-MEO and 2-LEO configuration ($\sigma_{e}=0.315$).

**Figure 4:**
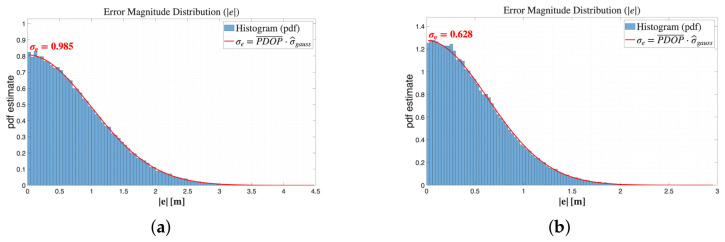
Positioning error PDFs under combined atmospheric delays and multipath effects: (**a**) 8-MEO configuration ($\sigma_{e}=0.985$) and (**b**) hybrid 6-MEO and 2-LEO configuration ($\sigma_{e}=0.628$).

In this section, we evaluate the positioning performance of the proposed LEO-PNT system compared to the traditional MEO constellation. A key aspect to highlight is that in order to isolate the impact of the characteristics of LEO satellites on positioning accuracy, the simulation results presented in Figure 2, Figure 3 and Figure 4 are based on a geometric configuration of the constellation that is kept strictly unchanged. The analysis starts from a crystallized snapshot of eight MEO satellites in known positions, which defines a baseline geometry for the user. Starting from this baseline constellation, in subsequent phases, rather than expanding the system by increasing the number of satellites, MEO satellites are progressively replaced with LEO ones, modifying only their operational altitude and transmission frequency; the spots to be replaced are randomly selected from the MEO constellation satellites. Due to this type of replacement, the system’s observation geometry matrix remains unchanged and, consequently, the Position Dilution of Precision (PDOP) remains constant across all evaluated configurations, in particular from the baseline configuration with eight MEO satellites to the hybrid configurations until the one with four MEOs and four LEOs. Thus, by eliminating PDOP variations, the system performance changes in terms of positioning error Probability Density Function and standard deviation, as shown in Figure 2, Figure 3 and Figure 4, can be attributed to the increase in pseudorange determination accuracy and consequently the implemented substitution directly translates into a variation in the pseudorange error, which ultimately impacts the accuracy of the user’s final position.

### 4.1. Ionospheric Delay

The total delay induced by the ionosphere along the signal propagation path is mainly determined by TEC [4]. In the case of Medium Earth Orbit systems, transmitting signals at the L1 frequency (1.575 GHz) [2,4,27], this atmospheric interference manifests itself as an error in the pseudorange measurement, which can be analytically modeled as [12]:

(1) $$\delta_{iono,MEO}=\frac{40.3}{f^{2}}TEC$$

In contrast, low Earth orbit satellites typically orbit at lower altitudes and transmit signals at higher frequencies (e.g., 12.5 GHz in the Ku-band) [4,10]. Because LEO satellites orbit within the ionosphere, signals transmitted to the ground only pass through the ionospheric layers below the satellite’s altitude and the partial electron content traversed by these signals is referred to as bottomside electron content ($TEC_{LEO}$) [25]. The adapted formula for the LEO ionospheric delay is given by:

(2) $$\delta_{iono,LEO}=\frac{40.3}{f^{2}}TEC_{LEO}$$

The TEC is calculated using the slant path theory based on the receiver’s position, the satellite elevation relative to the Earth’s surface and the VTEC (Vertical Total Electron Content), which is the electron content measured along a vertical line [28]. Based on the receiver’s position and the satellite elevation, the $TEC_{LEO}$ is a fraction of the total slant TEC; for this reason, in the following simulations, its value is set as 0.62·TEC. This fractional scaling of about 62% accurately represents a realistic scenario where LEO signal crosses the lower layers of the ionosphere. Recent studies, analyzing LEO navigation augmentation signals, demonstrate that the portion of bottomside electron content relative to total TEC ranges from 56% during night to 84% during daytime [5,25]. The chosen factor of 62% falls well within this experimentally validated range, producing a numerator factor of approximately 25 (∼40.3×0.62) compared to the full MEO equation.

The temporal evolution of ionospheric error is modeled using a Gauss–Markov process [5,29], with initial values $\delta_{iono,MEO}$ and $\delta_{iono,LEO}$ defining the delay distributions for each orbit [5]. Mitigation is handled by the Klobuchar model to minimize propagation errors for single-frequency users [19]. Simulations highlight a clear difference in ionospheric vulnerability between the two constellations. Specifically, LEO signals are assumed to only cross the bottomside of the ionosphere [25], leading to a proportional scaling of the Klobuchar correction. By comparing an 8-MEO baseline with an 8-LEO setup (Figure 2a,b), the positioning error PDFs demonstrate the intrinsic robustness of LEO signals due to their higher carrier frequency. The introduction of an LEO satellite into the constellation produces an improvement in terms of positioning error; the decrease in $\sigma_{e}$ depends on the lower impact that ionospheric transit has on a signal transmitted by an LEO, whose frequency is much higher than that of an MEO (Figure 2c). Increasing the number of LEO satellites (Figure 2d) results in a systematic reduction of the overall positioning error. As emerges from [30], adverse atmospheric conditions, such as the presence of ionospheric scintillations that compromise the synchronization phase with the receivers, and extreme space weather events, such as geomagnetic storms [31], can deteriorate the system’s performance; therefore, the possibility of having a greater number of visible satellites available, as obtained by combining MEO and LEO constellations, is fundamental to mitigate such effects and guarantee the accuracy and continuity of the service as required in safety-critical applications.

### 4.2. Tropospheric Delay

In contrast to MEO platforms, LEO satellites experience distinct tropospheric interactions due to their lower altitudes and rapid orbital dynamics. To account for these effects, the tropospheric delay is estimated using the Saastamoinen model [3], which utilizes meteorological parameters—Pressure ($P_{s}$), Temperature ($T_{s}$) and Humidity—to derive the hydrostatic (dry) and wet delay components [32]. The dry and wet components of the zenith delay are calculated as follows:

(3) $$d_{dry}=0.002277\cdot \frac{P_{s}}{f(\phi ,h)}$$

(4) $$d_{wet}=0.002277\cdot \frac{e_{s}\cdot \left(\frac{1255}{T_{s}}+0.05\right)}{f(\phi ,h)}$$

where ϕ is the receiver latitude, *h* is its altitude, and the gravitational scaling factor is defined as f(ϕ,h)=1−0.0266cos(2ϕ)−0.00028h. The partial pressure of water vapor $e_{s}$ is derived from the relative humidity [12,26]. Within the MATLAB environment, tropospheric error is characterized as a Gaussian distribution with bias. The total delay calculated through the Saastamoinen model serves as the mean of this distribution. To mitigate this effect, the deterministic component of the delay—estimated via the Saastamoinen model—is subtracted from the overall distribution to perform the necessary error correction [3]. The experimental results confirm that LEO satellite networks exhibit a remarkably lower vulnerability to tropospheric variations compared to higher orbits. The applied compensation strategy successfully eliminates the systematic bias from the error distribution, leaving only the stochastic zero-mean residual noise (Figure 3a,b). Furthermore, the study indicates that positioning precision improves in direct correlation with the density of the LEO constellation. By rapidly changing LEO elevation angles due to low-orbit satellites’ high angular velocity and employing deterministic bias compensation, the integration of LEO nodes provides a level of accuracy that standard MEO-only configurations cannot match in challenging environments. As seen in Figure 3c, the inclusion of even a single LEO satellite improves user positioning accuracy, due to its lower atmospheric delay profile. This enhancement becomes more pronounced as more LEO nodes are added; incorporating two LEO satellites (Figure 3d) enables substantial error mitigation, bringing the system’s standard deviation down to $\sigma_{e}=0.315$. This significant improvement allows the hybrid system to nearly match the performance benchmarks of an LEO-only architecture.

### 4.3. Multipath Error

LEO and MEO satellites are affected differently by the multipath effect, due to both signal characteristics and orbital geometry [9]. Multipath error exhibits strong temporal correlation, which depends on the dynamic geometry between the satellite, reflectors and the antenna.

According to the characterization in [6,20], multipath error is typically not zero-mean; instead, it manifests itself as time-varying biases whose statistical behavior is closely tied to the specific properties of the propagation channel. For this reason, to realistically simulate this behavior, the multipath error on a satellite is modeled as a Gauss–Markov process [26]. This stochastic model represents the temporal evolution of the disturbance as a time-correlated sequence rather than a simple white noise, where the current error state is functionally dependent on its preceding value. The process is regulated by a correlation time constant, which quantifies how quickly the reflection environment changes.

To ensure physical consistency of the simulated environment, multipath errors for MEO and LEO satellites are generated using separate time constants. Since multipath decorrelation depends on the satellite’s angular velocity relative to the receiver, the MEO satellites’ time constant is larger to represent a more stable reflection geometry; in contrast, a significantly shorter time constant is adopted for LEO satellites to model the rapid evolution of the reflection environment caused by their high orbital velocity. Within this setup, the distinct orbital characteristics of each constellation are explicitly considered to define the multipath time correlation: a constant of 300 s is set for MEO satellites to account for their stable multipath geometry, in line with established stochastic error models and ground processing standards [33,34,35]. A much shorter value of 30 s is applied to LEO satellites; this lower value first models the fast angular velocity and the consequent high-frequency variations in reflection conditions typical of low Earth orbits [18,36]. Furthermore, it is important to emphasize the fact that this time constant of 30 s for LEO satellites represents a conservative choice; as described in [18], the high angular velocity of LEOs can yield an improvement of up to 75% in decorrelating multipath errors compared to traditional GNSS constellations. For this reason, keeping the time constant at 30 s, slightly longer than the baseline decorrelation time actually allowed by the physical environment, ensures that the applied model does not overestimate the performance of the LEO satellite.

To mitigate multipath effects, a Geometry-Aided Smoothing variant of the Hatch filter is implemented [3,37]. The smoothed pseudorange ${\bar{\rho }}_{k}$ is computed as

(5) $${\bar{\rho }}_{k}=\alpha_{m}\rho_{raw,k}+\left(1-\alpha_{m}\right)\left({\bar{\rho }}_{k-1}+\Delta \rho \right)$$

where $\alpha_{m}$ is the smoothing coefficient, $\rho_{raw,k}$ is the raw measured pseudorange and Δρ is the geometric distance variation between time steps.

When integrating all three error sources (ionosphere, troposphere and multipath), the performance gap between MEO and hybrid constellations becomes even more pronounced. By introducing two LEO satellites into an MEO constellation (Figure 4b), the value of $\sigma_{e}$ is reduced from 0.985 (MEO-only constellation in Figure 4a) to 0.628, confirming a reduction of 36% in ground error. If the number of LEOs in the constellation increases, $\sigma_{e}$ is further reduced and consequently the precision of the final calculated position increases.

### 4.4. TLE Error

Unlike MEOs, low Earth orbit satellites do not directly transmit ephemerides and the orbital information necessary for pseudorange calculation. Instead, LEO satellites utilize Two-Line Elements (TLEs) provided by NORAD [38,39,40]. The pseudorange error contribution due to TLEs is modeled as a biased Gaussian distribution, applied only to LEO satellites. The model employs a standard deviation of 30 m alongside a mean value of 500 m, which represents the typical bias introduced by the use of TLEs in the determination of LEO orbits, considering a parameter update that was not earlier than 24 h ago, as shown in [23]. This offset is mainly due to the analytical simplifications introduced by the SGP4 (Simplified General Perturbations) propagator in modeling the forces acting on low Earth orbit satellites, primarily atmospheric drag, as demonstrated in [24]. Instead, the standard deviation of 30 m is consistent with the empirical validations performed using Global Positioning System (GPS) data and comparing them with the results obtained employing two-line elements [41]. As demonstrated in [42], decimeter-level accuracy is currently achieved in GPS-based Precise Orbit Determination by exploiting the post-processing of dual-frequency measurements; this result is achievable even in very low-orbit conditions where the impact of atmospheric drag is even more evident, as highlighted in [43]. However, LEO satellites, due to their smaller size compared to satellites orbiting at higher altitudes, can be equipped with onboard systems that are subject to stringent constraints in terms of size, weight and power consumption; these limitations also involve onboard computational resources. To account for these operational limitations and to ensure a conservative simulation scenario, a less stringent orbital accuracy is selected to model a possible alternative tracking technique to TLEs; for these reasons, a Gaussian distribution with a bias and a mean error of 100 m and a standard deviation of 1 m is adopted. This choice aims to demonstrate that an alternative technique with low complexity and low resource consumption, which can also be supported directly on board, is sufficient to reduce $\sigma_{e}$ and improve system performance with a consequent increase in user positioning precision.

**Figure 5:**
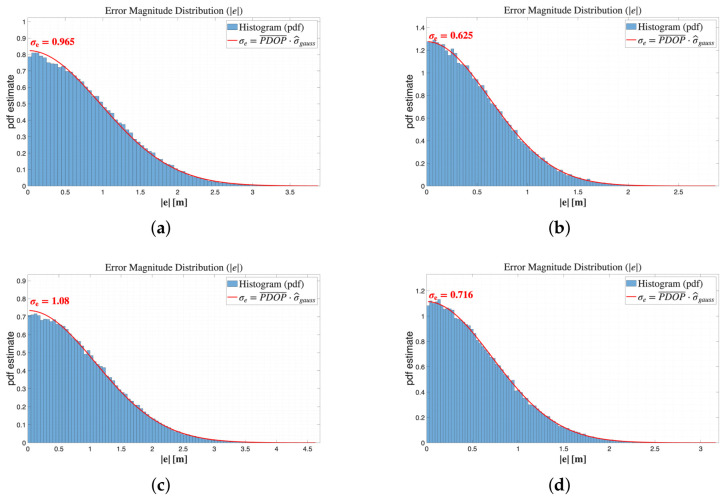
Positioning error PDFs under atmospheric and multipath errors isolating TLE uncertainty and clock bias: (**a**) TLE error with 1-LEO hybrid configuration ($\sigma_{e}=0.965$), (**b**) TLE error with 2-LEO hybrid configuration ($\sigma_{e}=0.625$), (**c**) clock error with 1-LEO hybrid configuration ($\sigma_{e}=1.08$) and (**d**) clock error with 2-LEO hybrid configuration ($\sigma_{e}=0.716$).

The analysis of TLE-related errors introduces a critical trade-off in LEO-augmented PNT systems. In contrast to atmospheric effects, where LEO satellites offer an inherent physical advantage, orbit determination acts as a temporary technological constraint. A significant finding of this study is that including a single LEO satellite into an MEO constellation does not immediately improve positioning accuracy if orbital uncertainty is elevated. As demonstrated in the findings, the 1-LEO configuration shows a $\sigma_{e}$ of 0.965 (Figure 5a), which is almost identical to the 8-MEO baseline (0.985). This occurs because the spatial bias caused by standard TLE data outweighs the benefits of a lower atmospheric delay and increased signal power. System performance improves significantly when the number of LEO satellites grows to two or more (Figure 5b); increasing the LEO count to two lowers $\sigma_{e}$ to 0.625, a 35% improvement. With multiple LEOs, the positioning filter is able to better distribute the residual orbital bias, allowing the atmospheric and multipath advantages to finally surpass the TLE uncertainty. The simulation confirms that by moving from the standard NORAD TLE to a more Precise Orbit Determination strategy, a performance jump is achievable.

### 4.5. Clock Error

In contrast to MEO constellations, LEO cannot have atomic clocks equipped, primarily due to the stringent volume constraints of small-satellite architectures and the energy requirements of such high-precision instruments [2,44]. This lack of a stable frequency standard introduces a temporal offset in pseudorange measurements, which averages approximately 100 ns [22]. As analyzed in [21], real LEO flight data indicate that even high-performance space-grade oscillators can accumulate milliseconds of clock bias over a two-week period if left unchecked, so adopting a 100 ns satellite clock bias in the simulation framework represents a realistic conservative scenario, reflecting the real-world rapid error growth of commercial quartz oscillators, generally utilized in LEO missions.

To represent the behavior of these oscillators, the clock bias is modeled as a Random Walk, a non-stationary stochastic process that accumulates uncertainty over time [21]. Consequently, at each discrete time step *k*, the frequency drift $d_{k}$ is defined as:

(6) $$d_{k}=d_{k-1}+\omega_{k}$$

where $\omega_{k}∼N\left(0,\sigma_{h_{mH}}^{2}\right)$ with $\sigma_{h_{mH}}=1$ ns. The total clock error $\epsilon_{CLK}\left(k\right)$ is obtained by superimposing a white Gaussian noise term to model instantaneous signal jitter [26]:

- Uncompensated parameters: a cumulative drift component combined with an instantaneous jitter $\eta_{inHJ}\left(k\right)$ with $\sigma_{inHJ}=60$ ns.
- Compensated parameters: error reduction is achieved by assuming a more stable reference and subtracting a correction term from the pseudorange.

The error introduced by the lack of a stable clock affects only the LEO satellites present in the constellation.

The analysis of integrating LEO satellites within MEO constellations demonstrates the direct impact of clock synchronization errors on user positioning accuracy. Experimental results indicate that the inclusion of a single LEO satellite maintains the standard deviation of the final positioning error, $\sigma_{e}$, at levels identical to those of a standalone MEO configuration. However, the integration of a second LEO satellite leads to a significant performance gain, achieving a value of $\sigma_{e}=0.716$. This occurs because increasing the number of LEOs also increases the number of satellites experiencing a smaller (and appropriately compensated) pseudorange error, thus benefiting from a reduction in the overall error. Combining multiple low-orbit satellites results in a lower pseudorange error capable of fully compensating for the higher error characteristic of MEOs (Figure 5d). The synergy of multiple low-orbit satellites yields a reduced pseudorange error, which effectively compensates for the inherent noise levels characteristic of traditional MEO constellations.

## 5. Discussion

### 5.1. Clock and TLE Error

**Figure 6:**
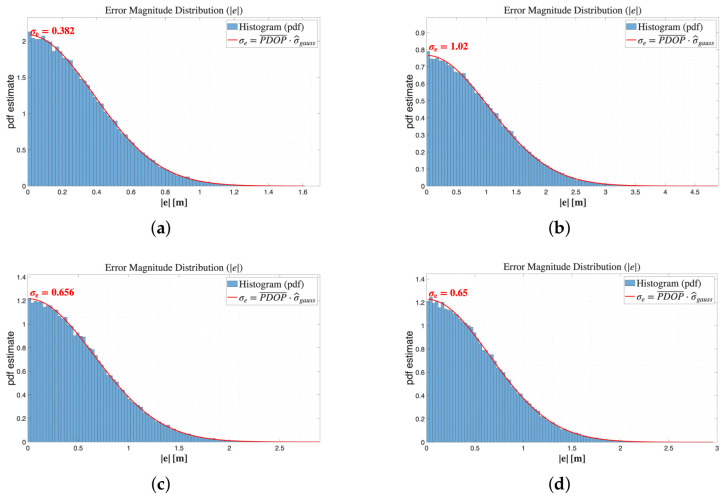
Positioning error PDFs for the cumulative error scenario (atmospheric, multipath, TLE and clock errors): (**a**) 8-LEO configuration ($\sigma_{e}=0.382$), (**b**) hybrid 7-MEO and 1-LEO configuration ($\sigma_{e}=1.020$), (**c**) hybrid 5-MEO and 3-LEO configuration ($\sigma_{e}=0.656$) and (**d**) hybrid 4-MEO and 4-LEO configuration ($\sigma_{e}=0.650$).

In this section, the cumulative effect of clock bias and TLE error is analyzed. If a single LEO satellite is introduced into an MEO constellation, the resulting PDF is shown in Figure 6b. As shown in previous analyses, and evident in Figure 6b, the positioning error $\sigma_{e}$ remains substantially unchanged compared to a configuration without LEO satellites.

To understand this behavior, it is necessary to decompose the total value to highlight how individual components contribute to the overall error based on separate analyses conducted in Section 4.1, Section 4.2, Section 4.3, Section 4.4 and Section 4.5. Since, as previously highlighted, the PDOP remains constant in the baseline and hybrid configurations, any variation in system performance does not result from geometric advantages, but is directly related to how the pseudorange errors change. As demonstrated in Section 4.3, the physical advantages of the signals transmitted by LEO satellites, particularly the reduced ionospheric and tropospheric delay and the rapid decorrelation of the multipath error, allow for a 40.9% improvement compared to a constellation of eight MEO satellites, going from $\sigma_{e}=0.985$ to 0.582. However, in the cumulative scenario with a single low Earth orbit satellite, these benefits are partially offset by the intrinsic space and power consumption limitations of the LEO satellite itself, namely the lack of an atomic clock, and the reduced precision in determining its position due to the use of TLEs for POD; for these reasons, the clock drift and the TLE orbital distortion are perfectly coupled, so the estimation algorithm is unable to distinguish the temporal error from the spatial uncertainty, causing the atmospheric and multipath advantages to be canceled out by the combined TLEs and clock noise. As the number of LEO satellites increases, the performance of the system improves significantly. With three LEOs (Figure 6c), the error standard deviation drops from 1.020 to 0.656, highlighting a 35% performance gain. To satisfy the error decomposition, this reduction is evaluated in the variance domain, where the total cumulative variance is mitigated from $1.040\;m^{2}$ to $0.430\;m^{2}$ ($\Delta \sigma^{2}=0.610\;m^{2}$). Observing three independent LEO satellites creates optimal spatial diversity for the least squares algorithm; this diversity enables spatial averaging, allowing the estimator to decouple and cancel TLE directional biases; by observing LEO satellites moving rapidly from different angles at successive epochs, the algorithm can average out errors due to the use of the TLEs and smooth out clock jitter through the redundancy of multiple LEO observations without changing the instantaneous PDOP. This mathematical decoupling solves the bottleneck and allows the system to benefit positively from the introduction of LEO satellites into the constellation. In particular, the results highlighted in Section 4.3 demonstrate that the vast majority of the variance reduction is due to the specific characteristics of the LEO signal ($\Delta \sigma^{2}=0.631\;m^{2}$), while the use of three low-orbit satellites allows the clock and TLE errors to be mitigated, such that their effect has a small contribution to the residual variance of only $0.091\;m^{2}$ ($0.656^{2}-0.582^{2}$), bringing the total cumulative error very close to what it would be if the LEO satellites were not affected by the two aforementioned errors.

An important aspect in the evaluation of the total error is to understand why a single LEO satellite in the constellation does not provide any improvement in the precision of the determined position. In this regard, the analysis must extend beyond the intrinsic limitations of low Earth orbit satellites and include the spatial and temporal dynamics of the observation geometry. From a spatial perspective, a single LEO satellite introduces a single observation equation into the estimation algorithm, linked to a single line-of-sight vector. Therefore, although an LEO signal has, by nature, better physical characteristics than a GNSS signal. The estimator is unable to fully benefit from the advantages deriving from the presence of the aforementioned satellite since the estimator itself operates on a matrix of observations where only one row is affected by the modification introduced in the constellation. In the absence of an adequate number of LEO satellites operating from different positions, the estimator does not have alternative spatial references required to triangulate and isolate error components along that single vector and, consequently, it cannot compensate and mitigate errors due to TLE and clock bias [45]. From a temporal perspective, LEO satellites move at high speeds compared to MEOs and complete an orbit in about 90 min. Consequently, the high orbital velocity means that the time interval during which a single LEO is visible above the user’s horizon is relatively short [45]. Due to this short time window, the least squares algorithm, which computes the navigation solution on an epoch-by-epoch basis, is unable to fully exploit the typical advantages of a low Earth orbit satellite; consequently, without an adequate number of supporting LEOs to sustain the spatial diversity over time, a single satellite thus acts as a transient and geometrically isolated observation that is overwhelmed by the errors of the remaining MEO part of the constellation.

To fully evaluate the improvement, four MEOs are replaced with low Earth orbit satellites. Taking into account all error sources, the results are shown in Figure 6d. The results indicate that the hybrid MEO-LEO architecture requires a critical threshold of LEO density to effectively suppress the cumulative error variance. The number of satellites to be replaced is reasonably set at three. When moving from three to four LEO satellites, system performance improvement encounters an error saturation phenomenon, as evidenced by the $\sigma_{e}$ values showing only a very small decrease in the performance parameter, from 0.656 with three LEOs to 0.650. This demonstrates a law of diminishing returns in the least squares estimator, and a three-LEO substitution achieves the minimum spatial diversity needed to decouple and distribute the TLE and clock errors across all three orthogonal spatial coordinates. Beyond this threshold, no tangible improvement can be achieved, as the addition of a fourth LEO still fails to reduce the residual error, which depends on the noise introduced by the remaining four MEO satellites in the constellation and on the thermal noise of the receiver. With 3 LEO, the algorithm has enough satellites in view from different angular positions to calculate and mitigate the TLE error components, so the fourth LEO does not add a new spatial dimension to the estimation geometry. Therefore, the optimal number of satellites to replace is reasonably set at three; a higher number would alter the balance of the hybrid constellation without producing any significant improvement in positioning accuracy.

### 5.2. Six-Satellite Constellation

**Figure 7:**
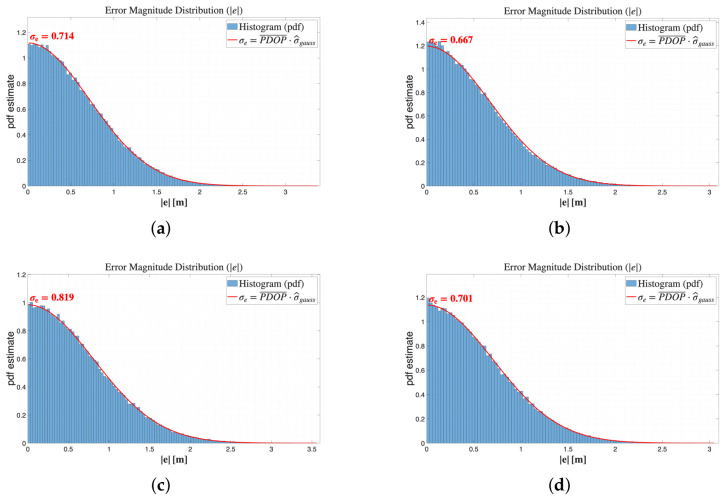
Positioning error PDFs for reduced constellations: (**a**) hybrid 5-MEO and 1-LEO configuration under overall error contributions ($\sigma_{e}=0.714$), (**b**) hybrid 4-MEO and 2-LEO configuration overall error contributions ($\sigma_{e}=0.667$), (**c**) 6-MEO configuration with atmospheric and multipath errors ($\sigma_{e}=0.819$) and (**d**) 6-MEO baseline plus 1-LEO satellite under overall error contributions ($\sigma_{e}=0.701$).

This section aims to analyze the behavior of the system when the constellation is composed of six satellites, evaluating how the introduction of LEO satellites can improve the accuracy of the positioning solution. As previously examined in the eight-satellite constellation, in this case as well, the system also benefits positively from the introduction of a low Earth orbit satellite, resulting in a reduction in overall error (Figure 7a). By increasing the number of replaced satellites, the accuracy of the solution improves, as seen with two LEOs in the constellation (Figure 7b). The error decreases with $\sigma_{e}$ equal to 0.667, although it does not reach the performance of an eight-satellite system (five MEOs and three LEOs). However, given the lower complexity of the current constellation, net of the slight difference in precision, we are working with a less complex system that can be profitably employed in applications that do not require extremely high accuracy.

Finally, it is interesting to evaluate the system’s behavior if an LEO is added to a six-MEO satellite system. A constellation of six MEO satellites in the presence of ionospheric, atmospheric and multipath error contributions yields the graph in Figure 7c. The introduction of an additional satellite (Figure 7d), moving in a low orbit, results in a reduction of positioning error, confirming that the presence of LEOs allows an effective improvement in system performance, even if the number of MEOs available to the receiver is reduced.

## 6. Conclusions

This research highlights that the combination of LEO constellations and traditional MEO-based GNSS represents a promising pathway for next-generation PNT systems. The simulation results suggest that a direct substitution of MEO satellites with LEO nodes does not inherently guarantee a reduction in positioning error. Indeed, our findings identify a specific performance trade-off: the integration of three LEO satellites provides an optimal balance between architectural complexity and accuracy. Beyond this threshold, the system exhibits a distinct error saturation mechanism. The modular architecture of the developed simulator ensures that these findings are not restricted to a 1D scenario, but are easily extendable to 2D and 3D coordinate systems without loss of generality. Despite these insights, certain research limitations must be acknowledged. First, although the analytical evaluation was conducted within a 1D vehicular framework, which represents a spatial simplification to precisely isolate longitudinal error components along a trajectory, the applied methodology remains inherently scalable. Second, the system’s robustness has not yet been simulated and validated in dynamic and highly perturbed environments typical of positioning systems. These may include intense multipath, spoofing, intentional jamming, adverse atmospheric conditions affecting pseudorange measurements, or degraded visibility scenarios where fewer than four satellites are in view, resulting in an overall reduction in system performance.

A fundamental finding of this work is that the intrinsic advantages of LEO platforms are effectively nullified by the absence of atomic clocks and high-precision orbit determination. To overcome these bottlenecks, future work must supplement specific technical implementation paths addressing key engineering challenges:

- Clock Dynamics: The adoption of quartz oscillators introduces significant non-stationary clock drift. Without robust compensation mechanisms, this drift remains a primary driver of error in the final positioning solution. The crucial engineering challenge is to develop miniaturized space-grade atomic clocks that can meet the stringent size, weight and power consumption constraints required for small LEO platforms, while ensuring the necessary stability and minimal drift essential for PNT applications.
- Ephemeris Accuracy: TLE-based ephemeris propagation is insufficient for high-precision PNT applications. Replacing TLEs with advanced Precise Orbit Determination algorithms is mandatory to exploit the LEO constellation’s potential. Future implementations will need to optimize real-time POD algorithms and this is possible, on the one hand, by overcoming the computational and power limitations of LEO on-board processors and, on the other, by employing satellite position determination techniques that use GNSS and by exploiting continuous inter-satellite links or advanced ground station uplinks to achieve and maintain centimeter-level orbit tracking, as well as by developing appropriate satellite orbit prediction models that take into account the actual forces acting in low Earth orbit.

The evolution toward a reliable hybrid GNSS/LEO architecture is strictly tied to overcoming these synchronization and orbital tracking bottlenecks. The development of space-grade miniaturized atomic clocks and advanced real-time orbital estimation techniques is essential not only for increasing accuracy, but also for ensuring the integrity of the system required by safety-critical sectors. This study provides an analytical foundation for future PNT solutions, offering a viable roadmap for autonomous driving and smart city infrastructures where standard GPS performance is no longer sufficient. 

## Figures and Tables

**Table 1:** Comparison of positioning error standard deviations ($\sigma_{e}$) across different orbital configurations under individual and combined error sources.

Error Source/Effect [m]	MEO-Only	Single-LEO	2-LEO	3-LEO
Individual Effects				
Ionospheric delay	0.802	0.775	0.605	0.555
Tropospheric delay	0.626	0.599	0.315	0.262
Combined Effects				
Multipath (+Iono, Tropo)	0.985 ^a^	0.844	0.628	0.582
TLE (+Iono, Tropo, Multipath)	–	0.965	0.625	0.594
Clock (+Iono, Tropo, Multipath)	–	1.080	0.716	0.685
All Errors Combined ^b^	0.985	1.020 ^c^	0.741	0.656

^a^ Value for 8 MEO satellites (0.819 for 6 MEO). ^b^ Evaluated with ionosphere, troposphere, multipath, TLE and clock errors. ^c^ Value for 1 LEO. Also tested: 5 MEO+1 LEO (0.714), 6 MEO+1 LEO (0.701) and 4 MEO + 2 LEO (0.667).

## Data Availability

The raw data supporting the conclusions of this article will be made available by the authors on request.
